# Insomnia symptom prevalence in England: a comparison of cross-sectional self-reported data and primary care records in the UK Biobank

**DOI:** 10.1136/bmjopen-2023-080479

**Published:** 2024-05-07

**Authors:** Melanie A de Lange, Rebecca C Richmond, Sophie V Eastwood, Neil M Davies

**Affiliations:** 1 MRC Integrative Epidemiology Unit, University of Bristol, Bristol, UK; 2 Population Health Sciences, Bristol Medical School, University of Bristol, Bristol, UK; 3 NIHR Oxford Health Biomedical Research Centre, University of Oxford, Oxford, UK; 4 Institute of Cardiovascular Science, University College London, London, UK; 5 Division of Psychiatry & Department of Statistical Sciences, University College London, London, UK; 6 K.G. Jebsen Center for Genetic Epidemiology, Department of Public Health and Nursing, Norwegian University of Science and Technology, Trondheim, Norway

**Keywords:** SLEEP MEDICINE, Electronic Health Records, Primary Health Care

## Abstract

**Abstract:**

**Objectives:**

We aimed to use a large dataset to compare self-reported and primary care measures of insomnia symptom prevalence in England and establish whether they identify participants with similar characteristics.

**Design:**

Cross-sectional study with linked electronic health records (EHRs).

**Setting:**

Primary care in England.

**Participants:**

163 748 UK Biobank participants in England (aged 38–71 at baseline) with linked primary care EHRs.

**Outcome measures:**

We compared the percentage of those self-reporting ‘usually’ having insomnia symptoms at UK Biobank baseline assessment (2006–2010) to those with a Read code for insomnia symptoms in their primary care records prior to baseline. We stratified prevalence in both groups by sociodemographic, lifestyle, sleep and health characteristics.

**Results:**

We found that 29% of the sample self-reported having insomnia symptoms, while only 6% had a Read code for insomnia symptoms in their primary care records. Only 10% of self-reported cases had an insomnia symptom Read code, while 49% of primary care cases self-reported having insomnia symptoms. In both primary care and self-reported data, prevalence of insomnia symptom cases was highest in females, older participants and those with the lowest household incomes. However, while snorers and risk takers were more likely to be a primary care case, they were less likely to self-report insomnia symptoms than non-snorers and non-risk takers.

**Conclusions:**

Only a small proportion of individuals experiencing insomnia symptoms have an insomnia symptom Read code in their primary care record. However, primary care data do provide a clinically meaningful measure of insomnia prevalence. In addition, the sociodemographic characteristics of people attending primary care with insomnia were consistent with those with self-reported insomnia, thus primary care records are a valuable data source for studying risk factors for insomnia. Further studies should replicate our findings in other populations and examine ways to increase discussions about sleep health in primary care.

**STRENGTHS AND LIMITATIONS OF THIS STUDY**

- The large sample size of this study meant that our estimates, even within strata, were very precise.
- The breadth of the electronic health record data meant we were also able to explore, validate and triangulate across multiple definitions of insomnia.
- By linking people’s primary care data to extensive self-report questionnaire data in the UK Biobank we were able to directly compare self-reported to primary-care indicated insomnia in the same population and explore the characteristics of those suffering from insomnia symptoms in great detail.
- The UK Biobank is not representative of the UK population. If having insomnia also affects participation in the UK Biobank, then this may have caused selection bias in our estimates of insomnia prevalence.
- Our definition of self-reported insomnia symptoms did not encompass early morning awakenings or impaired daytime function and therefore is not in line with established guidelines for insomnia diagnosis and treatment.

## Introduction

 Insomnia is a distressing, yet common, condition which has extensive consequences for population health.^1^ It has been associated with a variety of health problems including depression,^2 3^ substance use,^4 5^ dementia,^6^ diabetes^7^ and cardiovascular disease.^8 9^ In addition, insomnia has been associated with lower productivity^10^ and higher absenteeism in the workplace,^11 12^ higher accident rates,^11 13^ greater healthcare utilisation^11 12^ and reduced quality of life.^12 14^

Estimates of insomnia prevalence differ depending on the definition used. A review of 50 studies from different countries found that around a third of the general population have insomnia symptoms (defined as difficulty initiating/maintaining sleep or non-restorative sleep, regardless of the underlying cause). In addition, 9%–15% suffer from daytime consequences of insomnia, 8%–18% are dissatisfied with their sleep and 6% meet the criteria for an insomnia diagnosis.^15^ However, most previous studies estimating insomnia prevalence rely on participants self-reporting their symptoms and diagnoses through questionnaires or telephone interviews. Responses may therefore be subject to recall bias.^16^ Many surveys have used small sample sizes consequently limiting their precision.^17^ Selection bias could also be an issue in these studies if survey non-response was also related to insomnia prevalence.^18^

In recent decades there has been a rapid growth in the use of electronic health records (EHRs) in population health research.^19^ EHRs potentially offer larger sample sizes, rich longitudinal data, lower risk of recall bias and in countries such as the UK (where 98% of the population is registered with a primary care doctor and consultations are free of charge^20^), can reduce selection bias.^19^ However, to date, EHR research on insomnia prevalence is limited. One US study of 15 family practices (n=7928) found that 9.4% of primary care patients had an insomnia diagnosis, 7.4% had been prescribed an insomnia-related medication and 3.9% had both a diagnosis and prescription. Diagnoses and prescriptions were greater in women than men, and increased with age.^21^ Another study found that 15% of a sample of 440 000 US Veterans had a prescription for an insomnia medication, while 6% had a diagnosis for insomnia.^22^

By definition, EHRs only capture events where a patient visits a healthcare professional. As a result, mild or temporary conditions may be missed.^22^ This is particularly likely to be the case for insomnia where only around a third of those self-reporting insomnia symptoms also self-report seeking help from a healthcare provider for them.^23^,^25^ Only a few studies have explored this gap in data capture by comparing people’s self-reported insomnia symptoms to their actual medical records. One study in Majorca found that of patients who met the criteria for an insomnia diagnosis during a telephone survey (n=99), only 40% had a consultation for insomnia and only 12% had an insomnia diagnosis in their medical record. Another study of 5 UK GP practices (n=327) found that while 34% of patients self-reported insomnia symptoms, only 19% of this group had a primary care consultation for insomnia or a mood problem in the following 12 months and 30% had a consultation or prescription for insomnia/mood problem.^26^ These studies suggest that EHRs are only picking up a small proportion of people experiencing insomnia symptoms. However, the generalisability of this research is limited due to small sample sizes and their results are yet to be replicated in larger studies. It is therefore not clear how useful EHRs are in measuring the prevalence of insomnia.

Using UK Biobank data, which combines self-reported measures of insomnia with linked primary care records for over 160 000 people in England, this study aimed to compare self-reported and primary care measured insomnia symptom prevalence. We also aimed to establish whether self-report and primary care insomnia data identify participants with similar characteristics in order to evaluate the value of EHRs in measuring insomnia prevalence.

## Methods

### Study population

The UK Biobank is a population-based cohort study of around 500 000 adults who were aged 39–69 when recruited from across the UK between 2006 and 2010 (participation rate: 5.5%).^27^ It contains comprehensive questionnaire data, as well as physical measurements and biological samples.^27^ Linked primary care data is also available for around 45% of UK Biobank participants.^28^

**Figure 1:**
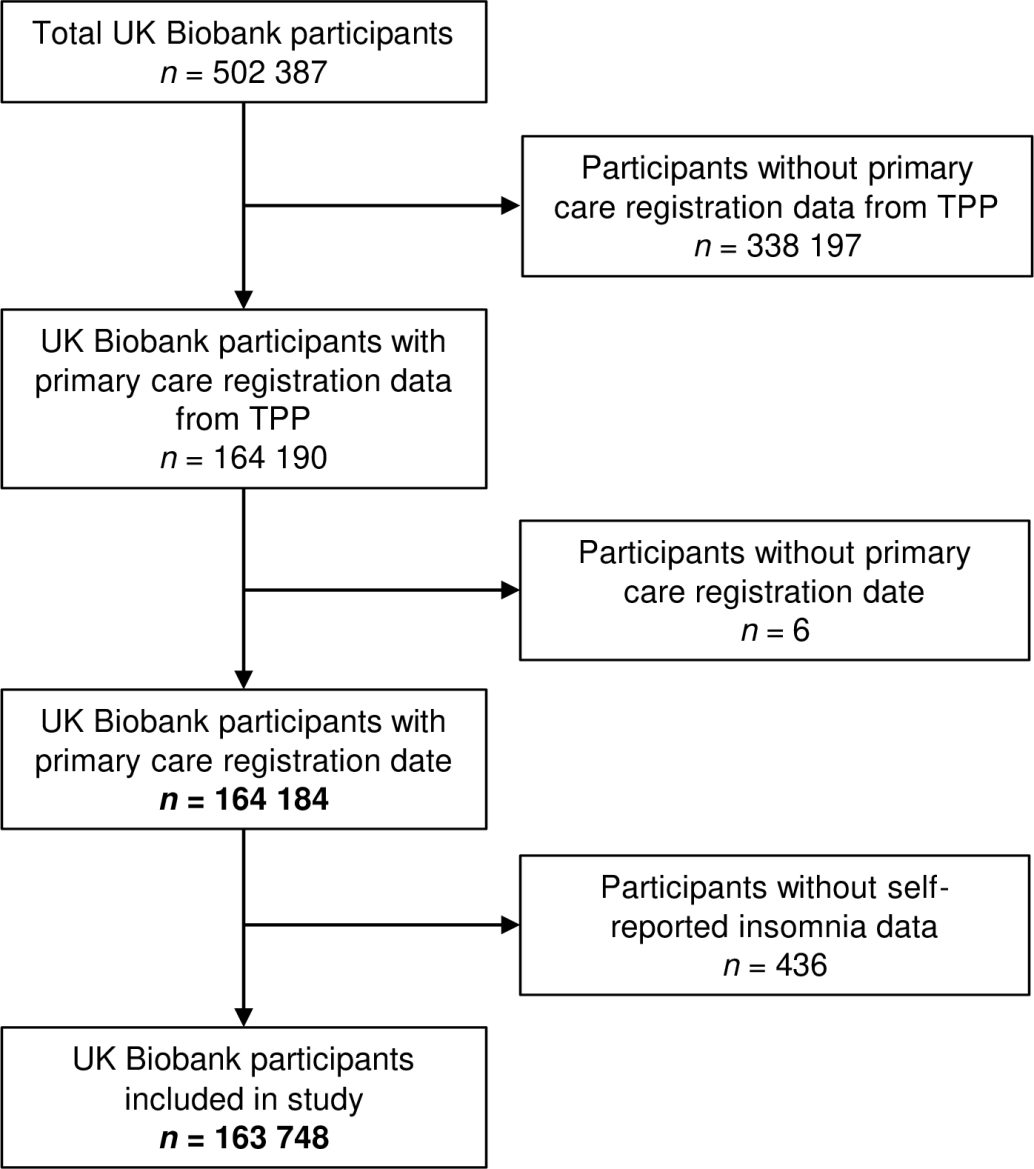
Participant flow diagram.

Of the 502 387 participants, we removed 338 197 who did not have linked primary care registration data provided by TPP (an England-only dataset). We also removed 6 participants who did not have a registration date in their primary care registration data and 436 participants without data on self-reported insomnia. This gave us a sample of 163 748 participants (see figure 1).

### Insomnia symptom case identification

#### Self-reported insomnia symptoms cases

Participants were asked in a UK Biobank touchscreen questionnaire: ‘Do you have trouble falling asleep at night or do you wake up in the middle of the night?’. If they pressed the help button they were told: ‘If this varies a lot, answer this question in relation to the last 4 weeks’. Options for participants’ answers were: ‘Never/rarely’, ‘Sometimes’, ‘Usually’ and ‘Prefer not to answer’. We coded ‘Prefer not to answer’ as missing. Participants were counted as a self-reported insomnia symptoms case if they answered ‘Usually’.

#### Primary care insomnia symptoms cases

Participants were designated as having primary care insomnia symptoms if they had a Read code for insomnia symptoms in their primary care record on or prior to UK Biobank baseline assessment (2006–2010). Insomnia Read codes occurring after baseline were excluded from our analysis in order to be consistent with the self-report question, which asked about existing symptoms. Read codes for events outside the date of an individual’s registration period at their GP practice (or practices) were excluded as coverage of symptoms during these periods may be unreliable. Sensitivity analyses defined insomnia by Read code and concomitant hypnotic use (see sensitivity analyses section).

#### Code list creation

To create our list of Read codes for insomnia symptoms we searched the UK Biobank Read CTV3 code lookup table for codes containing the following strings: *insomn* *sleep* or *wak*. This gave us an initial list of 385 insomnia Read codes. This list was then refined by primary care clinician review. We kept our list of insomnia symptom Read codes broad so that we would capture anyone who answered ‘Usually’ to the non-specific UK Biobank self-report question (which asked about insomnia symptoms regardless of the cause). We therefore included codes involving physical (organic) causes of sleep problems (eg, asthma, chronic obstructive pulmonary disease, sleep apnoea), parasomnias (eg, sleepwalking) and sleep pattern disturbances. Duplicate codes were removed. This resulted in a final list of 181 Read codes (online supplemental table S1). Many Read terms for insomnia do not differentiate between diagnoses and symptoms, therefore we did not examine the prevalence of diagnoses alone.^29^

We mapped the BNF (British National Formulary) prescription codes in the TPP UK Biobank primary care data to the standard BNF codes produced by the NHS Business Services Authority. The first six digits of the TPP BNF codes correspond to the first six digits of standard BNF codes (these relate to the BNF chapter, section and paragraph).^28^ We therefore reformatted the TPP BNF codes in our dataset to consist of six-digit codes. We defined a prescription for insomnia symptoms as a prescription for a hypnotic medication (six digit BNF code: 040101) (online supplemental table S2).

### Covariates

To ascertain the characteristics of participants with insomnia symptoms identified by the self-report data and primary care records, we included a number of sociodemographic (age, sex, ethnic group, household income, Index of Multiple Deprivation, current employment status, highest qualification, household size, living with spouse/partner and home area population density), sleep (duration, chronotype, snoring, dozing, napping, how easy find getting up in the morning, night shift work), lifestyle (physical activity, tea/coffee intake, smoking, alcohol intake, risk taking) and health (body mass index, menopause, depression, worrying, overall health rating) factors in our analysis. Further details on how the covariates were handled are provided in online supplemental method.

### Statistical analysis

We calculated the prevalence of insomnia symptoms in both the self-reported and primary care data. We also identified the proportion of self-reported insomnia symptom cases that were primary care insomnia symptom cases and vice versa. To identify and compare the characteristics of self-reported and primary care insomnia symptom cases, insomnia prevalence was stratified by sociodemographic, lifestyle, sleep and health variables, and visualised in coefficient plots. Analyses were performed in Stata V.16 via JupyterLab in DNA Nexus. Full code is available online (https://github.com/MeldeLange/insomnia-comparison-study).

### Sensitivity analyses

To explore the effect of using different definitions of insomnia symptoms in primary care on the number of cases and their overlap with self-reported insomnia symptom cases, we performed two sensitivity analyses. First, we looked at the timing of symptoms, defining primary care cases as only those with a Read code in the 12 months or 4 weeks prior to UK Biobank baseline assessment. Second, we defined primary care cases as those with a hypnotic prescription prior to baseline or those with a Read code and concomitant prescription for hypnotics within 90 days of the Read code.

### Patient and public involvement

This study was a secondary analysis of existing data. Patients and the public were not involved in the design, conduct, reporting or dissemination plans. However, the UK Biobank routinely disseminates study results to its participants and the public via its website (http://www.ukbiobank.ac.uk/news/), social media channels (@uk_biobank) and press releases. UK Biobank also holds an annual conference highlighting recent research which is available to watch on its website.

## Results

### Participant characteristics and insomnia symptom prevalence

Characteristics of the study population, overall and stratified by self-reported or primary care insomnia symptoms case, are presented in table 1 (see online supplemental table S3 for full participant characteristics). In this study 45% of participants were male, 62% were aged 55 or over and 75% had a sleep duration of 7 hours or more. We found that 29% of the sample self-reported having insomnia symptoms, while only 6% had a primary care Read code for insomnia symptoms.

**Table 1:** Characteristics of total sample and groups stratified by insomnia symptoms status

	Total sample	Self-report insomnia symptoms case		Primary care insomnia symptoms case	
		No	Yes	No	Yes
**Total, % (n**)	100% (163 748)	71.1% (116 414)	28.9% (47 334)	94.0% (153 919)	6.0% (9829)
**Variables, % (n**)					
Sex					
Male	45.4% (74 422)	48.5% (56 417)	38.0% (18 005)	45.8% (70 537)	39.5% (3885)
Age					
Under 45	10.0% (16 427)	11.2% (13 008)	7.2% (3419)	10.2% (15 685)	7.5% (742)
45–54	27.8% (45 580)	28.6% (33 329)	25.9% (12 251)	28.0% (43 064)	25.6% (2516)
55–64	42.8% (70 082)	41.6% (48 415)	45.8% (21 667)	42.6% (65 606)	45.5% (4476)
65 or over	19.3% (31 659)	18.6% (21 662)	21.1% (9997)	19.2% (29 564)	21.3% (2095)
Ethnic group					
White	94.8% (154 707)	94.3% (109 461)	95.9% (45 246)	94.8% (145 451)	94.5% (9256)
Mixed	0.5% (838)	0.5% (580)	0.5% (258)	0.5% (786)	0.5% (52)
Asian/Asian British	2.4% (3905)	2.6% (3045)	1.8% (860)	2.4% (3661)	2.5% (244)
Black/Black British	1.3% (2045)	1.4% (1621)	0.9% (424)	1.3% (1922)	1.3% (123)
Chinese	0.3% (414)	0.3% (335)	0.2% (79)	0.3% (393)	0.2% (21)
Other	0.8% (1297)	0.8% (977)	0.7% (320)	0.8% (1201)	1.0% (96)
Average household income (before tax)					
<£18 000	24.7% (34 400)	22.5% (22 453)	30.1% (11 947)	24.3% (31839)	31.2% (2561)
£18 000–£30 999	26.5% (36 868)	26.2% (26 136)	27.0% (10 732)	26.4% (34 633)	27.2% (2235)
£31 000–£51 999	25.7% (35 759)	26.5% (26 391)	23.6% (9368)	25.8% (33 864)	23.1% (1895)
£52 000–£100 000	18.6% (25 898)	19.6% (19 580)	15.9% (6318)	18.8% (24 645)	15.3% (1253)
>£100 000	4.6% (6462)	5.1% (5091)	3.5% (1371)	4.7% (6202)	3.2% (260)
Index of Multiple Deprivation for England quartiles					
Q1 (0.76–7.85)	25.0% (39 626)	25.7% (28 866)	23.5% (10 760)	25.1% (37 374)	23.5% (2252)
Q2 (7.86–13.59)	25.1% (39 684)	25.4% (28 553)	24.3% (11 131)	25.1% (37 374)	24.1% (2310)
Q3 (13.6–23.85)	25.0% (39 491)	25.0% (28 076)	24.9% (11 415)	25.0% (37 172)	24.2% (2319)
Q4 (23.86–81.59)	24.9% (39 435)	24.0% (26 981)	27.2% (12 454)	24.7% (36 746)	28.1% (2689)
Body mass index					
Underweight	0.5% (805)	0.5% (554)	0.5% (251)	0.5% (752)	0.5% (53)
Healthy weight	31.8% (51 756)	32.6% (37 725)	29.8% (14 031)	32.0% (49 018)	28.1% (2738)
Overweight	42.7% (69 517)	43.4% (50 215)	41.0% (19 302)	42.9% (65 697)	39.1% (3820)
Obese	25.0% (40 748)	23.6% (27 286)	28.6% (13 462)	24.6% (37 600)	32.3% (3148)
Sleep duration					
3–4 hours	1.1% (1824)	0.2% (264)	3.3% (1560)	1.0% (1460)	3.8% (364)
5–6 hours	23.5% (38 163)	16.6% (19 284)	40.5% (18 879)	22.8% (34 848)	34.3% (3315)
7–8 hours	67.4% (109 653)	74.4% (86 234)	50.2% (23 419)	68.3% (104 406)	54.2% (5247)
9 or more hours	8.0% (12 989)	8.8% (10 178)	6.0% (2811)	8.0% (12 239)	7.8% (750)

We found that only 10% of self-reported insomnia symptom cases were also a primary care insomnia symptom case. Meanwhile, only 49% of primary care insomnia symptom cases were also a self-reported case (table 2).

**Table 2:** Cross-tabulation of primary care and self-reported insomnia symptom cases

Primary care insomnia symptom case		Self-reported insomnia symptom case		
		No	Yes	Total
No	Frequency	111 392	42 527	153 919
	Row %	72.4	27.6	100.0
	Column %	95.7	89.8	94.0
Yes	Frequency	5022	4807	9829
	Row %	51.1	48.9	100.0
	Column %	4.3	10.2	6.0
Total	Frequency	116 414	47 334	163 748
	Row %	71.1	28.9	100.0
	Column %	100.0	100.0	100.0

### Characteristics of self-reported and primary care insomnia symptom cases

Onlinesupplemental table S4 and figures S1S4 show that participants who self-reported insomnia symptoms and those with insomnia Read codes in their primary care records had similar characteristics. Sociodemographic correlates of being a primary care or self-reported insomnia symptoms case included being female, older, in the lowest household income category (<£18 000), in the highest quartile of deprivation, in the ‘other’ employment status category, with no qualifications, living in a one-person household, not living with a spouse/partner and not living in a rural area.

In addition, reporting a sleep duration of 3–4 hours, being a definite evening or definite morning chronotype, ‘often’ dozing during the day, ‘usually’ napping during the day, finding getting up in the morning ‘not at all easy’ and not doing shift work were also characteristics of those reporting insomnia or having a primary care record indicating insomnia.

Lifestyle characteristics of primary care or self-reported insomnia symptoms cases included being in the lowest physical activity quartile (metabolic equivalent task minutes/week), drinking 0–1 or 6+ cups of coffee per day, drinking 6+ cups of tea per day, being a previous or current smoker, and drinking alcohol less than once a month. Furthermore, health correlates of self-reported and primary care-recorded insomnia symptoms cases included women having been through the menopause, being underweight or obese, having experienced a depressed mood nearly every day in the past 2 weeks, being a worrier and rating your overall health as poor.

There were a few noticeable differences between the characteristics of self-reported and primary care insomnia symptom cases. Being a snorer was a correlate of being a primary care insomnia symptoms case, but being a non-snorer was a correlate of self-reporting insomnia. In addition, describing yourself as a risk taker was a characteristic of those having a primary care record indicating insomnia, whereas the opposite was true for self-reported cases. Differences in terms of ethnicity were also observed: being mixed or white was a correlate of being a self-reported case, whereas being in the ‘other’ category was a correlate of being a primary care case.

### Sensitivity analyses

Prevalence of primary care measured insomnia symptoms decreased from 6% to 0.1% of our sample when our definition of a primary care symptom case changed from having had an insomnia Read code prior to baseline to having had an insomnia Read code in the 4 weeks prior to baseline (table 3). It also fell to 1.9% when our definition of a primary care case required having a Read code prior to baseline and being prescribed a hypnotic within 90 days of the Read code, and fell further to 0.03% when we required a Read code in the 4 weeks prior to baseline and being prescribed a hypnotic within 90 days of the Read code. The prevalence of being prescribed a hypnotic medication prior to baseline was higher than the prevalence of having an insomnia Read code prior to baseline (11% vs 6%).

**Table 3:** Prevalence of primary care measured insomnia symptoms cases and overlap with self-reported insomnia symptom cases according to different definitions of a primary care insomnia symptoms case

Definition of primary care symptom case	% of total sample	% of self-reported cases that are primary care cases	% of primary care cases that are self-reported cases
Prescription for hypnotic prior to baseline	11.3	17.1	43.6
Read code prior to baseline (main analysis)	6	10.2	48.9
Read code in 12 months prior to baseline	1.2	2.3	55.8
Read code in 4 weeks prior to baseline	0.1	0.3	63.6
Read code prior to baseline and prescription for hypnotic within 90 days of Read code	1.9	3.7	57.7
Read code in 12 months prior to baseline and prescription for hypnotic within 90 days of Read code	0.3	0.8	65.0
Read code in 4 weeks prior to baseline and prescription for hypnotic within 90 days of Read code	0.03	0.08	72.6

As the strictness of our definition of a primary care insomnia symptom case increased, the proportion of primary care cases that were also self-reported cases (the specificity) increased and the proportion of self-reported cases that were also primary care cases decreased (the sensitivity) (see online supplemental tables S5–S10 for full cross-tabulations).

## Discussion

We used data from the UK Biobank to compare the prevalence of self-reported insomnia symptoms to the prevalence indicated by linked primary care records. We included all insomnia symptoms and sleep disturbance, regardless of the underlying cause.

Insomnia symptoms were common: 29% of our sample self-reported having frequent insomnia symptoms. This finding is highly consistent with a previous review of 50 studies from different countries, which estimated that around a third of the general population report having insomnia symptoms.^15^ In this study, only 6% of participants’ primary care records contained Read codes for insomnia symptoms. This is slightly lower than expected given that previous studies estimate that 6% of the general population meets the clinical criteria for an insomnia diagnosis^15^ and our study of symptoms was broader in scope. This inconsistency may be due to previous studies reporting insomnia diagnosis prevalence from interviews, whereas we measured the proportion of people who had acted on their symptoms and reported them to a primary care doctor. Our estimate is also slightly lower than a previous study by Klingman and Sprey^21^ which found the prevalence of insomnia diagnoses alone in primary care records in the USA to be 9%.^21^ This difference could be due to the difference in sample sizes (n=163 748 vs n=7928), cultural differences in visiting a health practitioner for sleep-related issues, sample selection bias in either sample or true differences in the rates of insomnia in the two populations.

In our sample, 1.9% of participants had a Read code for insomnia symptoms and a prescription for a hypnotic medication within 90 days of that Read code. Klingman and Sprey^21^ reported a 4% prevalence of insomnia in the USA with both diagnosis and prescription codes in primary care records. However, that study did not restrict when the medication was prescribed. Our finding that 11% of our sample had been prescribed a hypnotic medication was slightly higher than Klingman and Sprey’s prevalence of 7.6%. This could be due to cultural differences in prescribing practices and the availability of non-pharmaceutical treatments. The fact that we found prevalence of hypnotic prescriptions to be higher than the prevalence of insomnia symptom Read codes may be due to practitioners prescribing these drugs for conditions other than insomnia (such as agitation in dementia/psychotic disease or as a muscle relaxant for back pain) or prescribing them while documenting insomnia in the free text notes rather than recording a Read code.

We found that only 10% of people self-reporting insomnia symptoms had a primary care Read code for insomnia symptoms. This provides further evidence that only a small proportion of people experiencing insomnia seek help from a healthcare professional,^22^,^26^ and suggests that EHRs only capture a small proportion of those experiencing insomnia symptoms. As these are likely to be the most severe cases, or those not responsive to self-management, this may lead to amplification bias in associations between insomnia and health outcomes such as cardiovascular disease.

The low level of help-seeking behaviour for insomnia may be because people perceive insomnia as something that is harmless, trivial or amenable to self-management.^30 31^ In addition, stigma may deter people from seeking help^30^,^32^ or they may be unaware of the treatment options for insomnia, or concerned about the effectiveness and safety of sleeping tablets.^30^ In England, although referral for cognitive behavioural therapy for insomnia (CBT-I) is recommended as a first line treatment when insomnia symptoms are unlikely to resolve soon, its availability is limited.^33 34^ Consequently, many people rely on self-help remedies such as reading, listening to music and relaxation, or use over-the-counter or complementary medicines to aid sleep.^23 24 31^ However, new digital treatments for insomnia, such as NICE-approved Sleepio, could help to expand the treatment options available to primary care doctors.^33^

Surprisingly, we found that only 49% of primary care insomnia symptom cases also self-reported having insomnia symptoms. Possible explanations for this include that we looked at people’s primary care records from birth until they entered the UK Biobank study. It is possible that people may have experienced insomnia and visited their doctor a long time ago, then subsequently experienced an improvement in their symptoms before self-reporting their symptoms at the time of study entrance. This is supported by the fact that when we only included those with insomnia Read codes in the 4 weeks before baseline in our sensitivity analyses, the proportion of primary care insomnia symptom cases that self-reported insomnia symptoms rose (to 64% or 73% for those with a Read code 4 weeks prior to baseline and prescription within 90 days of the Read code). It is also possible that people with insomnia did not self-report having symptoms because they were ameliorated by medication or due to the stigma attached to having insomnia.

We found that the characteristics of self-reported and primary care-defined insomnia symptom cases were remarkably similar. Following previous studies,^15 21^ we found that key correlates of being a primary care or a self-reported insomnia symptom case were being female, older, not living with a partner, having lower educational attainment and incomes, and having poorer physical and mental health. We also found that women who had been through menopause, not living in a rural area, having an extreme chronotype, being less physically active, drinking lots of tea or coffee, and smoking were predictors of primary care and self-reported insomnia symptom cases. These consistent findings suggest that primary care records can provide valuable evidence about population level risk factors for insomnia. They could also be clinically important for GPs as some of the characteristics identified (eg, tea drinking/exercise) are modifiable lifestyle factors.

We also found that snoring predicted being a primary care insomnia symptoms case. However, the opposite was true of self-reported cases (ie, people who self-reported having insomnia were less likely to report snoring). This suggests that snoring may be a key risk factor for prompting those with insomnia to visit their primary care doctor to discuss their sleep. The discrepancy in snoring between self-report and primary care-identified insomnia cases may be due to the fact that the UK Biobank question about snoring actually relates to participants’ partners’ report of their snoring (‘Does your partner or a close relative or friend complain about your snoring?’). Because snoring can affect the partner’s sleep^35^ and mental health,^36^ their partner may encourage them to seek medical help, even if they themselves do not recognise that they snore. However, this was not supported by our analysis which suggests that primary care insomnia cases are more likely to be living alone. An alternative explanation is the fact snoring can be a sign of obstructive sleep apnoea, and that insomnia and obstructive sleep apnoea often occur together. For example, 40%–60% of those with sleep apnoea also have insomnia symptoms. This has led to the identification of a new disorder: ‘comorbid insomnia and obstructive sleep apnoea' (COMISA). However, more research is needed to fully understand this disorder.^37^ It may be that those with insomnia are more likely to be diagnosed with this by a GP if they also have sleep apnoea because sleep apnoea itself is more likely to impair daytime functioning, such as presenting with daytime drowsiness and difficulty driving, so GPs are more likely to diagnose and treat it.

Strengths of our study include the large sample size, which meant our estimates, even within strata, were very precise. In addition, the extensive, detailed self-report questionnaire combined with linked EHR data allowed us to explore, validate and triangulate across multiple definitions of insomnia. Our study also has several limitations. First, the UK Biobank is not representative of the UK population, with participants more likely to be female, healthier, older and live in less socioeconomically deprived areas than non-participants.^27^ If having insomnia also affects participation in the UK Biobank, then this may have caused selection bias in our estimates of insomnia prevalence. This is possible, as our study only included a small proportion of ethnic minority participants, and ethnic minorities are more likely to experience sleep problems and may experience socioeconomic disadvantage, itself a correlate of insomnia symptoms.^38^

A further limitation is the lack of a Gold Standard measure of insomnia. In this study, insomnia prevalence differed depending on the primary care definition used and our estimates differed from those of previous research, which again may be due to differences in definition. This makes it difficult to compare the prevalence of insomnia across populations. The prevalence of insomnia cases was extremely low when we placed time restrictions on insomnia Read codes or when a concomitant hypnotic prescription was required. This suggests that the most constructive measure of insomnia in primary care data is having a Read code for insomnia symptoms alone, at any point throughout a person’s medical history. It should also be noted that the self-report insomnia question asked in the UK Biobank did not encompass early morning awakenings or impaired daytime function. Our definition of self-reported insomnia symptoms is therefore not in line with established guidelines for insomnia diagnosis and treatment.^39^ We used a very broad list of Read codes to define insomnia symptoms in primary care. A narrower Read code list would produce a lower prevalence of primary care insomnia symptoms, as well as a smaller overlap between primary care and self-report insomnia symptom cases.

This study used routinely collected health data which was not collected for research purposes. The quality of primary care data relies on the accuracy (eg, data entry errors/wrong codes) and completeness (ie, recording of all diagnoses/symptoms) of inputted insomnia Read codes. This may vary between GP practices and practitioners, between health conditions, and over time. Studies have found that recording of subjective diagnoses (such as insomnia) is less consistent than that of more objective diagnoses.^40^ It is possible that even when patients discuss insomnia symptoms with their GP they are not recorded correctly and this could have contributed to the low prevalence of insomnia symptoms found in this study.

In conclusion, this study found that in a large sample, primary care symptom codes only capture a small proportion of those experiencing insomnia symptoms in the population. As these are likely to be the most extreme cases, associations between insomnia and other health outcomes may be amplified in primary care data. Nonetheless, EHRs provide a valuable data source for studying insomnia, offering clinically meaningful measures of insomnia prevalence, objective insights into severe insomnia, large sample sizes and longitudinal data. Furthermore, the relationships observed between insomnia symptoms and sociodemographic characteristics were consistent in both self-report and primary care datasets. Consequently, researchers exploring population-level risk factors for insomnia are likely to draw similar conclusions using either dataset. Further studies should replicate our findings in other populations and examine the best ways to increase discussions about sleep health in primary care.

## Supplementary material

10.1136/bmjopen-2023-080479 online supplemental file 1

10.1136/bmjopen-2023-080479 online supplemental file 2

10.1136/bmjopen-2023-080479 online supplemental file 3

10.1136/bmjopen-2023-080479 online supplemental file 4

10.1136/bmjopen-2023-080479 online supplemental file 5

10.1136/bmjopen-2023-080479 online supplemental file 6

10.1136/bmjopen-2023-080479 online supplemental file 7

## Data Availability

Data may be obtained from a third party and are not publicly available.
