# Supplementary material for: Insomnia symptom prevalence in England: a comparison of cross-sectional self-reported data and primary care records in the UK Biobank
Source: BMJ Open. 2024 May 7;14(5):e080479. doi: 10.1136/bmjopen-2023-080479 (PMC11086527; doi:10.1136/bmjopen-2023-080479)
Supplement: online supplemental file 1 [file bmjopen-2023-080479supp001.pdf]

Table S1: Insomnia Symptoms Read Code List

Read Code	Description
1B1B.	insomnia (& c/o)
1B1B0	initial insomnia
1B1B1	middle insomnia
1B1B2	late insomnia
3148.	sleep studies - procedure
663N.	asthma disturbing sleep
663N1	asthma disturbs sleep weekly
663N2	asthma disturbs sleep frequently
E274.	non-organic sleep disorders (& [hypersomnia] or [insomnia])
E2740	unspecified non-organic sleep disorder
E2741	insomnia: [transient] or [nos]
E2742	persistent insomnia
E2747	sleepwalking
E2748	sleep terrors
E274A	sleep drunkenness
E274B	repeated rapid eye movement sleep interruptions
E274C	other sleep stage or arousal dysfunction
E274D	(repetitive intrusions of sleep) or (restless sleep)
E274E	"short-sleeper"
E274F	reversed sleep-wake cycle
E274y	(dreams) or (other non-organic sleep disorder)
E274z	non-organic sleep disorder nos
Eu510	nonorganic insomnia
Eu512	[x] nonorganic disorder of the sleep-wake schedule: [psychogenic inversion of circadian rhythm (including nyctohemeral rhythm and sleep rhythm)]
Eu51y	[x]other nonorganic sleep disorders
Eu51z	[x]nonorganic sleep disorder, unspecified
Fy00.	disorders of initiating and maintaining sleep
Fy03.	sleep apnoea (& [obstructive])
Fyu58	[x]other sleep disorders
K5A21	menopausal sleeplessness
R005.	[d]sleep disturbances
R0050	[d]sleep disturbance, unspecified
R0051	[d]insomnia with sleep apnoea
R0052	[d]insomnia nos
R0053	([d]hypersomnia with sleep apnoea) or (sleep apnoea syndrome)
R0055	[d]sleep rhythm inversion
R0056	[d]sleep rhythm irregular
R0057	[d]sleep-wake rhythm non-24-hour cycle
R0058	[d]sleep dysfunction with sleep stage disturbance
R0059	[d]sleep dysfunction with arousal disturbance
R005z	[d]sleep dysfunction nos
TJ7z0	adverse reaction to sleeping pill nos
TK02.	[x]overdose - sleeping tabs
U1A2.	[x]accidental poisoning with sleeping tablets
U202.	[x]overdose - sleeping tabs
Ua15W	ability to sleep
Ua1FM	sleep and rest interventions
Ua1ZQ	sleep talking
X003N	familial fatal insomnia
X007q	sleep-wake disorder
X007s	insomnia nos
X007u	difficulty getting to sleep

X007v	difficulty in sleep maintenance
X0083	sleep apnoea
X0084	obstructive sleep apnoea
X0085	central sleep apnoea
X0086	mixed sleep apnoea
X0087	alveolar sleep apnoea
X0088	transient sleep-wake rhythm disorder
X0089	delayed sleep phase syndrome
X008A	non-24 hour sleep-wake cycle
X008C	sleep-related head banging
X008D	sleep-related painful erections
X764D	low level of awareness whilst sleep walking
X764E	low level of reactivity whilst sleep walking
X764F	low level of motor skill whilst sleep walking
X764G	blank, staring face whilst sleep walking
X764H	unresponsive to communication whilst sleep walking
X764I	not easily wakened from sleep walking
X764J	no recollection of sleep walk
X764K	sleep automatism
X769S	wakefulness
X76AE	sleep rhythm problem
X76AF	cannot sleep at all
X76AG	not getting enough sleep
X76AJ	wakes and cannot sleep again
X76AK	wakes early
X76AL	circumstances interfere with sleep
X76AM	symptoms interfere with sleep
X76AN	restless sleep
X76AO	unrefreshed by sleep
X77f4	sleep latency test
X77f5	full sleep study
X77f6	mini-sleep study
XE0ux	insomnia (& symptom) or somnolence
XE1Yg	transient insomnia
XE1Yi	repetitive intrusions of sleep
XE1Yj	other non-organic sleep disorder
XE1Zr	non-organic disorder of the sleep-wake schedule
XE1bI	sleep disorders (& [insomnia] or [nightmares] or [sleepwalking (& somnambulism)])
XE1gP	[d]sleep dysfunction nec
XE2Pv	insomnia
XE2Q5	non-organic sleep disorder
XE2cd	[d]sleep disturbances (& [hypersomnia] or [insomnia])
XE2nU	[d]hypersomnia with sleep apnoea
XM06i	sleep disturbance
XM06j	irregular sleep-wake pattern
XM06k	disorder of sleep-wake cycle
XM0CT	c/o - insomnia
XM0Go	appliance for sleep apnoea
XM0yu	[d]insomnia
XSGLz	light sleep
Xa7wV	difficulty sleeping
XaEGP	[d]sleep apnoea syndrome
XaFqr	poor sleep pattern
XaIp8	sleep management
XaIti	delayed onset of sleep
XaJKk	sleep studies - specialty
XaKv8	chronic obstructive pulmonary disease disturbs sleep
XaMs7	sleep studies nec

XaN5q	seen in sleep clinic
XaO8S	pittsburgh sleep quality index
XaO8T	insomnia severity index
XaOb7	referral to sleep clinic
XaP4v	sleep hygiene behaviour education
XaP4w	sleep restriction therapy
XaYGN	able to sleep with sedation
Xaag2	on melatonin for sleep disorder
XabE4	symptom assessment scale - difficulty sleeping score
XafMO	signposting to the sleep council
ZV1B1	[v]personal history of unhealthy sleep-wake schedule
c88A.	sleepia 50mg capsule
c88G.	vantage pharmacy sleep aid 50mg tablet
c88H.	care night time sleep aid 25mg tablet
iz1E.	natrasleep tablet
x02kT	natrasleep
x03mt	sleepia
x05t9	vantage pharmacy sleep aid
.1B1B	insomnia (& symptom) or somnolence
.1B1Q	poor sleep pattern
.1BX0	delayed onset of sleep
.1BX9	light sleep
.38D0	pittsburgh sleep quality index
.38D1	insomnia severity index
.663N	asthma disturbing sleep
.66Yg	chronic obstructive pulmonary disease disturbs sleep
.8G99	sleep restriction therapy
.8G9B	sleep hygiene behaviour education
.8HTn	referral to sleep clinic
.8Q0.	sleep management
.9Nk0	seen in sleep clinic
.E4A.	sleep disorders (& [insomnia] or [nightmares] or [sleepwalking (& somnambulism)])
.E4A0	sleep terrors
.E4A1	wakes early
.H66.	sleep apnoea
.R05.	[d]sleep disturbances (& [hypersomnia] or [insomnia])
.R050	[d]sleep disturbance, unspecified
.R051	[d]insomnia with sleep apnoea
.R052	[d]insomnia
.R053	[d]hypersomnia with sleep apnoea
.R055	[d]sleep rhythm inversion
.R056	[d]sleep rhythm irregular
.R057	[d]sleep-wake rhythm non-24-hour cycle
.R058	[d]sleep dysfunction with sleep stage disturbance
.R059	[d]sleep dysfunction with arousal disturbance
.R05Z	[d]sleep dysfunction nec
1B1Q.	poor sleep pattern
1BX0.	delayed onset of sleep
38D0.	pittsburgh sleep quality index
38D1.	insomnia severity index
38Da.	berlin questionnaire for sleep apnoea
66Yg.	chronic obstructive pulmonary disease disturbs sleep
70658	sleep studies - procedure
7065A	sleep studies nec
745A2	mini-sleep study
7P1B0	full sleep study
8G99.	sleep restriction therapy
8G9B.	sleep hygiene behaviour education

8HTn.	referral to sleep clinic
8Q0..	sleep management
9Ngt.	on melatonin for sleep disorder
9Nk0.	seen in sleep clinic
9b9Y.	sleep studies - specialty
Eu51.	non-organic sleep disorder
Eu513	sleepwalking
Eu514	sleep terrors
Fy0..	sleep-wake disorder
Fy02.	disorder of sleep-wake cycle
H5B..	sleep apnoea
H5B0.	obstructive sleep apnoea
X007t	nonorganic insomnia
