# Supplementary material for: Insomnia symptom prevalence in England: a comparison of cross-sectional self-reported data and primary care records in the UK Biobank
Source: BMJ Open. 2024 May 7;14(5):e080479. doi: 10.1136/bmjopen-2023-080479 (PMC11086527; doi:10.1136/bmjopen-2023-080479)
Supplement: online supplemental file 2 [file bmjopen-2023-080479supp002.pdf]

**Table S2: List of hypnotic drugs**

| **Hypnotics included under six digit BNF code 040101** |
|---|
| Melatonin |
| Toquilone |
| Chloral hydrate |
| Clomethiazole |
| Flunitrazepam |
| Flurazepam |
| Loprazolam |
| Lormetazepam |
| Nitrazepam |
| Temazepam |
| Zaleplon |
| Triclofos sodium |
| Zolpidem |
| Zopiclone |
