# Supplementary material for: Insomnia symptom prevalence in England: a comparison of cross-sectional self-reported data and primary care records in the UK Biobank
Source: BMJ Open. 2024 May 7;14(5):e080479. doi: 10.1136/bmjopen-2023-080479 (PMC11086527; doi:10.1136/bmjopen-2023-080479)
Supplement: online supplemental file 4 [file bmjopen-2023-080479supp004.pdf]

TABLE S3 Characteristics of Total Sample and Groups Stratified by Insomnia Symptoms Status

	Total Sample	Self-report insomnia symptoms case		Primary care insomnia symptoms case	
		No	Yes	No	Yes
**Total, % (n)**	100% (163,748)	71.1% (116,414)	28.9% (47,334)	94.0% (153,919)	6.0% (9,829)
**Variables, % (n)**					
Male	45.4% (74,422)	48.5% (56,417)	38.0% (18,005)	45.8% (70,537)	39.5% (3,885)
Age					
Under 45	10.0% (16,427)	11.2% (13,008)	7.2% (3,419)	10.2% (15,685)	7.5% (742)
45-54	27.8% (45,580)	28.6% (33,329)	25.9% (12,251)	28.0% (43,064)	25.6% (2,516)
55-64	42.8% (70,082)	41.6% (48,415)	45.8% (21,667)	42.6% (65,606)	45.5% (4,476)
65 or over	19.3% (31,659)	18.6% (21,662)	21.1% (9,997)	19.2% (29,564)	21.3% (2,095)
Ethnic group					
White	94.8% (154,707)	94.3% (109,461)	95.9% (45,246)	94.8% (145,451)	94.5% (9,256)
Mixed	0.5% (838)	0.5% (580)	0.5% (258)	0.5% (786)	0.5% (52)
Asian/Asian British	2.4% (3,905)	2.6% (3,045)	1.8% (860)	2.4% (3,661)	2.5% (244)
Black/Black British	1.3% (2,045)	1.4% (1,621)	0.9% (424)	1.3% (1,922)	1.3% (123)
Chinese	0.3% (414)	0.3% (335)	0.2% (79)	0.3% (393)	0.2% (21)
Other	0.8% (1,297)	0.8% (977)	0.7% (320)	0.8% (1,201)	1.0% (96)
Average household income (before tax)					
<£18,000	24.7% (34,400)	22.5% (22,453)	30.1% (11,947)	24.3% (31,839)	31.2% (2,561)
£18,000-£30,999	26.5% (36,868)	26.2% (26,136)	27.0% (10,732)	26.4% (34,633)	27.2% (2,235)
£31,000-£51,999	25.7% (35,759)	26.5% (26,391)	23.6% (9,368)	25.8% (33,864)	23.1% (1,895)
£52,000-£100,000	18.6% (25,898)	19.6% (19,580)	15.9% (6,318)	18.8% (24,645)	15.3% (1,253)
>£100,000	4.6% (6,462)	5.1% (5,091)	3.5% (1,371)	4.7% (6,202)	3.2% (260)
Index of Multiple Deprivation for England Quartiles					
Q1 (0.76-7.85)	25.0% (39,626)	25.7% (28,866)	23.5% (10,760)	25.1% (37,374)	23.5% (2,252)
Q2 (7.86-13.59)	25.1% (39,684)	25.4% (28,553)	24.3% (11,131)	25.1% (37,374)	24.1% (2,310)
Q3 (13.6-23.85)	25.0% (39,491)	25.0% (28,076)	24.9% (11,415)	25.0% (37,172)	24.2% (2,319)
Q4 (23.86-81.59)	24.9% (39,435)	24.0% (26,981)	27.2% (12,454)	24.7% (36,746)	28.1% (2,689)
Current employment status					
Paid employment / self-employed	56.3% (91,752)	59.1% (68,514)	49.3% (23,238)	56.8% (87,095)	47.6% (4,657)
Retired	34.7% (56,594)	33.1% (38,330)	38.7% (18,264)	34.4% (52,806)	38.7% (3,788)
Other	9.0% (14,718)	7.8% (9,067)	12.0% (5,651)	8.7% (13,386)	13.6% (1,332)
Highest qualification					
None	18.2% (29,457)	16.7% (19,173)	22.0% (10,284)	18.0% (27,321)	22.0% (2,136)
College/University degree	31.0% (50,109)	32.6% (37,476)	27.0% (12,633)	31.2% (47,475)	27.2% (2,634)
A/AS levels or equivalent	10.7% (17,327)	11.0% (12,675)	9.9% (4,652)	10.7% (16,350)	10.1% (977)
O levels/GCSEs or equivalent	21.9% (35,390)	21.6% (24,846)	22.5% (10,544)	21.8% (33,226)	22.3% (2,164)
CSEs or equivalent	5.9% (9,502)	5.8% (6,697)	6.0% (2,805)	5.9% (8,941)	5.8% (561)
NVQ/HND/HNC or equivalent	7.1% (11,436)	7.0% (8,106)	7.1% (3,330)	7.1% (10,777)	6.8% (659)
Other professional qualifications	5.3% (8,613)	5.3% (6,078)	5.4% (2,535)	5.3% (8,056)	5.7% (557)
Household size					
1 person	18.7% (30,323)	17.8% (20,620)	20.7% (9,703)	18.4% (28,197)	21.8% (2,126)
2 people	48.7% (79,181)	47.7% (55,214)	51.0% (23,967)	48.6% (74,320)	49.9% (4,861)
3-5 people	31.4% (51,012)	33.1% (38,217)	27.2% (12,795)	31.6% (48,377)	27.1% (2,635)
6 or more people	1.3% (2,071)	1.4% (1,581)	1.0% (490)	1.3% (1,959)	1.2% (112)
Live with spouse/partner	90.1% (119,229)	90.3% (85,848)	89.6% (33,381)	90.2% (112,525)	88.0% (6,704)
Home area population density					
Urban	83.3% (134,995)	83.1% (95,729)	83.8% (39,266)	83.4% (126,909)	82.8% (8,086)
Town	9.5% (15,424)	9.5% (10,930)	9.6% (4,494)	9.5% (14,411)	10.4% (1,013)

Rural	7.2% (11,585)	7.4% (8,490)	6.6% (3,095)	7.2% (10,923)	6.8% (662)
Primary care insomnia case	6.0% (9,829)	4.3% (5,022)	10.2% (4,807)	0.0% (0)	100.0% (9,829)
Self-report insomnia case	28.9% (47,334)	0.0% (0)	100.0% (47,334)	27.6% (42,527)	48.9% (4,807)
Sleep duration					
3-4 hours	1.1% (1,824)	0.2% (264)	3.3% (1,560)	1.0% (1,460)	3.8% (364)
5-6 hours	23.5% (38,163)	16.6% (19,284)	40.5% (18,879)	22.8% (34,848)	34.3% (3,315)
7-8 hours	67.4% (109,653)	74.4% (86,234)	50.2% (23,419)	68.3% (104,406)	54.2% (5,247)
9 or more hours	8.0% (12,989)	8.8% (10,178)	6.0% (2,811)	8.0% (12,239)	7.8% (750)
Chronotype					
Definite morning	24.5% (40,046)	24.4% (28,348)	24.8% (11,698)	24.5% (37,509)	25.9% (2,537)
Morning more than evening	31.9% (51,985)	32.2% (37,322)	31.1% (14,663)	32.0% (49,102)	29.4% (2,883)
No preference	10.6% (17,334)	10.8% (12,494)	10.3% (4,840)	10.6% (16,329)	10.3% (1,005)
Evening more than morning	25.3% (41,229)	25.4% (29,433)	25.0% (11,796)	25.2% (38,724)	25.6% (2,505)
Definite evening	7.7% (12,590)	7.2% (8,394)	8.9% (4,196)	7.6% (11,719)	8.9% (871)
Snore	37.6% (57,300)	38.4% (41,725)	35.9% (15,575)	37.5% (53,720)	39.7% (3,580)
Doze/fall asleep during the day when don’t mean to					
Never/rarely	75.8% (123,494)	77.6% (89,897)	71.4% (33,597)	76.1% (116,544)	71.3% (6,950)
Sometimes	21.3% (34,716)	20.4% (23,620)	23.6% (11,096)	21.1% (32,376)	24.0% (2,340)
Often	2.8% (4,641)	2.0% (2,281)	5.0% (2,360)	2.7% (4,183)	4.7% (458)
All of the time	0.0% (1)	0.0% (0)	0.0% (1)	0.0% (1)	0.0% (0)
Nap during the day					
Never/rarely	55.8% (91,166)	56.8% (66,032)	53.2% (25,134)	56.1% (86,168)	50.9% (4,998)
Sometimes	38.9% (63,559)	38.5% (44,713)	39.9% (18,846)	38.7% (59,441)	42.0% (4,118)
Usually	5.3% (8,728)	4.7% (5,439)	7.0% (3,289)	5.2% (8,033)	7.1% (695)
How easy find getting up in morning					
Not at all easy	4.0% (6,556)	2.8% (3,234)	7.0% (3,322)	3.8% (5,818)	7.5% (738)
Not very easy	13.7% (22,430)	12.0% (13,969)	17.9% (8,461)	13.5% (20,655)	18.1% (1,775)
Fairly easy	49.2% (80,446)	50.9% (59,167)	45.1% (21,279)	49.5% (75,987)	45.5% (4,459)
Very easy	33.0% (53,930)	34.3% (39,795)	29.9% (14,135)	33.3% (51,101)	28.9% (2,829)
Job involves night shift work					
Never/rarely	95.0% (155,418)	94.7% (110,170)	95.6% (45,248)	94.9% (146,032)	95.5% (9,386)
Sometimes	2.8% (4,573)	3.0% (3,437)	2.4% (1,136)	2.8% (4,321)	2.6% (252)
Usually	0.8% (1,320)	0.9% (1,021)	0.6% (299)	0.8% (1,257)	0.6% (63)
Always	1.4% (2,331)	1.5% (1,705)	1.3% (626)	1.4% (2,207)	1.3% (124)
Metabolic Equivalent TasK (MET) minutes per week quartiles					
Q1 (0-813)	25.0% (32,985)	24.0% (22,771)	27.6% (10,214)	24.8% (30,802)	28.5% (2,183)
Q2 (815-1815)	25.0% (32,907)	25.3% (23,992)	24.1% (8,915)	25.0% (31,002)	24.8% (1,905)
Q3 (1816.8-3679)	25.0% (32,900)	25.4% (24,079)	23.9% (8,821)	25.1% (31,146)	22.9% (1,754)
Q4 (3679.2-19278)	25.0% (32,919)	25.2% (23,890)	24.4% (9,029)	25.1% (31,090)	23.8% (1,829)
Coffee intake					
0-1 cups/day	48.9% (79,880)	48.6% (56,417)	49.7% (23,463)	48.8% (74,916)	50.6% (4,964)
2-3 cups/day	30.9% (50,434)	31.2% (36,259)	30.0% (14,175)	30.9% (47,495)	30.0% (2,939)
4-5 cups/day	13.7% (22,378)	13.8% (16,071)	13.4% (6,307)	13.8% (21,115)	12.9% (1,263)
6 or more cups/day	6.5% (10,645)	6.3% (7,373)	6.9% (3,272)	6.5% (10,007)	6.5% (638)
Tea intake					
0-2 cups/day	39.9% (65,146)	39.9% (46,322)	39.9% (18,824)	39.9% (61,334)	38.9% (3,812)
3-5 cups/day	40.7% (66,509)	41.2% (47,875)	39.5% (18,634)	40.7% (62,552)	40.4% (3,957)
6-8 cups/day	15.5% (25,247)	15.1% (17,582)	16.2% (7,665)	15.4% (23,677)	16.0% (1,570)
9 or more cups/day	4.0% (6,458)	3.8% (4,382)	4.4% (2,076)	3.9% (5,997)	4.7% (461)
BMI					
Underweight	0.5% (805)	0.5% (554)	0.5% (251)	0.5% (752)	0.5% (53)
Healthy weight	31.8% (51,756)	32.6% (37,725)	29.8% (14,031)	32.0% (49,018)	28.1% (2,738)
Overweight	42.7% (69,517)	43.4% (50,215)	41.0% (19,302)	42.9% (65,697)	39.1% (3,820)
Obese	25.0% (40,748)	23.6% (27,286)	28.6% (13,462)	24.6% (37,600)	32.3% (3,148)

Takes risks	26.8% (42,123)	27.2% (30,430)	25.7% (11,693)	26.7% (39,528)	27.6% (2,595)
Smoking status					
Never	54.8% (89,408)	56.1% (65,087)	51.6% (24,321)	55.1% (84,450)	50.7% (4,958)
Previous	35.0% (57,079)	34.0% (39,390)	37.5% (17,689)	34.9% (53,463)	37.0% (3,616)
Current	10.2% (16,646)	9.9% (11,511)	10.9% (5,135)	10.1% (15,449)	12.3% (1,197)
Alcohol intake frequency					
Daily/almost daily	20.0% (32,726)	19.9% (23,191)	20.2% (9,535)	20.1% (30,891)	18.7% (1,835)
3-4 times a week	23.1% (37,800)	23.9% (27,791)	21.2% (10,009)	23.3% (35,787)	20.5% (2,013)
Once or twice a week	26.0% (42,560)	26.4% (30,738)	25.0% (11,822)	26.1% (40,153)	24.5% (2,407)
1-3 times a month	11.2% (18,364)	11.1% (12,865)	11.6% (5,499)	11.2% (17,199)	11.9% (1,165)
Special occasions only	11.4% (18,594)	10.8% (12,547)	12.8% (6,047)	11.2% (17,225)	14.0% (1,369)
Never	8.3% (13,558)	7.9% (9,174)	9.3% (4,384)	8.2% (12,536)	10.4% (1,022)
Have had menopause (women only)	76.1% (64,905)	72.4% (41,509)	83.6% (23,396)	75.7% (60,234)	82.2% (4,671)
Frequency depressed mood past 2 weeks category					
Not at all	76.1% (118,835)	80.5% (89,626)	65.3% (29,209)	76.8% (112,731)	66.0% (6,104)
Several days	18.5% (28,886)	15.7% (17,518)	25.4% (11,368)	18.1% (26,596)	24.8% (2,290)
More than half the days	3.3% (5,098)	2.5% (2,830)	5.1% (2,268)	3.2% (4,633)	5.0% (465)
Nearly every day	2.1% (3,253)	1.2% (1,345)	4.3% (1,908)	1.9% (2,862)	4.2% (391)
Are a worrier	56.4% (89,744)	51.6% (58,247)	68.2% (31,497)	55.7% (83,381)	66.4% (6,363)
Overall health rating					
Excellent	14.9% (24,332)	16.9% (19,584)	10.1% (4,748)	15.3% (23,509)	8.4% (823)
Good	58.2% (94,846)	60.7% (70,349)	52.1% (24,497)	58.7% (89,952)	50.0% (4,894)
Fair	22.0% (35,870)	19.3% (22,434)	28.6% (13,436)	21.5% (32,954)	29.8% (2,916)
Poor	4.9% (7,927)	3.1% (3,581)	9.2% (4,346)	4.4% (6,778)	11.7% (1,149)
