# Supplementary material for: Insomnia symptom prevalence in England: a comparison of cross-sectional self-reported data and primary care records in the UK Biobank
Source: BMJ Open. 2024 May 7;14(5):e080479. doi: 10.1136/bmjopen-2023-080479 (PMC11086527; doi:10.1136/bmjopen-2023-080479)
Supplement: online supplemental file 5 [file bmjopen-2023-080479supp005.pdf]

TABLE S4 Self-reported and primary care insomnia symptom cases stratified by sociodemographics, lifestyle, sleep & health characteristics

	**Self-reported insomnia case**	**Primary care insomnia case**
Row %		
Sex		
Female	32.8	6.7
Male	24.2	5.2
Age		
Under 45	20.8	4.5
45-54	26.9	5.5
55-64	30.9	6.4
65 or over	31.6	6.6
Ethnic group		
White	29.2	6.0
Mixed	30.8	6.2
Asian/Asian British	22.0	6.2
Black/Black British	20.7	6.0
Chinese	19.1	5.1
Other	24.7	7.4
Average household income (before tax)		
<£18,000	34.7	7.4
£18,000-£30,999	29.1	6.1
£31,000-£51,999	26.2	5.3
£52,000-£100,000	24.4	4.8
>£100,000	21.2	4.0
Index of Multiple Deprivation for England Quartiles		
Q1 (0.76-7.85)	27.2	5.7
Q2 (7.86-13.59)	28.0	5.8
Q3 (13.6-23.85)	28.9	5.9
Q4 (23.86-81.59)	31.6	6.8
Current employment status		
Paid employment / self-employed	25.3	5.1
Retired	32.3	6.7
Other	38.4	9.1
Highest qualification		
None	34.9	7.3
College/University degree	25.2	5.3
A/AS levels or equivalent	26.8	5.6
O levels/GCSEs or equivalent	29.8	6.1
CSEs or equivalent	29.5	5.9
NVQ/HND/HNC or equivalent	29.1	5.8
Other professional qualifications	29.4	6.5
Household size		
1 person	32.0	7.0
2 people	30.3	6.1
3-5 people	25.1	5.2
6 or more people	23.7	5.4
Live with spouse/partner		
No	29.7	7.0
Yes	28.0	5.6

Home area population density		
Urban	29.1	6.0
Town	29.1	6.6
Rural	26.7	5.7
Sleep duration		
3-4 hours	85.5	20.0
5-6 hours	49.5	8.7
7-8 hours	21.4	4.8
9 or more hours	21.6	5.8
Chronotype		
Definite morning	29.2	6.3
Morning more than evening	28.2	5.5
No preference	27.9	5.8
Evening more than morning	28.6	6.1
Definite evening	33.3	6.9
Snore		
No	29.3	5.7
Yes	27.2	6.2
Doze/fall asleep during the day when don't mean to		
Never/rarely	27.2	5.6
Sometimes	32.0	6.7
Often	50.9	9.9
All of the time	100.0	0.0
Nap during the day		
Never/rarely	27.6	5.5
Sometimes	29.7	6.5
Usually	37.7	8.0
How easy find getting up in morning		
Not at all easy	50.7	11.3
Not very easy	37.7	7.9
Fairly easy	26.5	5.5
Very easy	26.2	5.2
Job involves night shift work		
Never/rarely	29.1	6.0
Sometimes	24.8	5.5
Usually	22.7	4.8
Always	26.9	5.3
Metabolic Equivalent TasK (MET) minutes per week quartiles		
Q1 (0-813)	31.0	6.6
Q2 (815-1815)	27.1	5.8
Q3 (1816.8-3679)	26.8	5.3
Q4 (3679.2-19278)	27.4	5.6
Coffee intake		
0-1 cups/day	29.4	6.2
2-3 cups/day	28.1	5.8
4-5 cups/day	28.2	5.6
6 or more cups/day	30.7	6.0
Tea intake		
0-2 cups/day	28.9	5.9
3-5 cups/day	28.0	5.9
6-8 cups/day	30.4	6.2
9 or more cups/day	32.1	7.1
BMI		
Underweight	31.2	6.6
Healthy weight	27.1	5.3

Overweight	27.8	5.5
Obese	33.0	7.7
Takes risks		
No	29.3	5.9
Yes	27.8	6.2
Smoking status		
Never	27.2	5.5
Previous	31.0	6.3
Current	30.8	7.2
Alcohol intake frequency		
Daily/almost daily	29.1	5.6
3-4 times a week	26.5	5.3
Once or twice a week	27.8	5.7
1-3 times a month	29.9	6.3
Special occasions only	32.5	7.4
Never	32.3	7.5
Have had menopause (women only)		
No	22.5	5.0
Yes	36.0	7.2
Frequency depressed mood past 2 weeks		
Not at all	24.6	5.1
Several days	39.4	7.9
More than half the days	44.5	9.1
Nearly every day	58.7	12.0
Are a worrier		
No	21.2	4.6
Yes	35.1	7.1
Overall health rating		
Excellent	19.5	3.4
Good	25.8	5.2
Fair	37.5	8.1
Poor	54.8	14.5
