# Supplementary material for: Insomnia symptom prevalence in England: a comparison of cross-sectional self-reported data and primary care records in the UK Biobank
Source: BMJ Open. 2024 May 7;14(5):e080479. doi: 10.1136/bmjopen-2023-080479 (PMC11086527; doi:10.1136/bmjopen-2023-080479)

Figures S1-4 Coefficient Plots

FIGURE S1 Coefficient plot of primary care and self-reported insomnia symptom cases stratified by sex, age, ethnicity, household income, deprivation and employment status

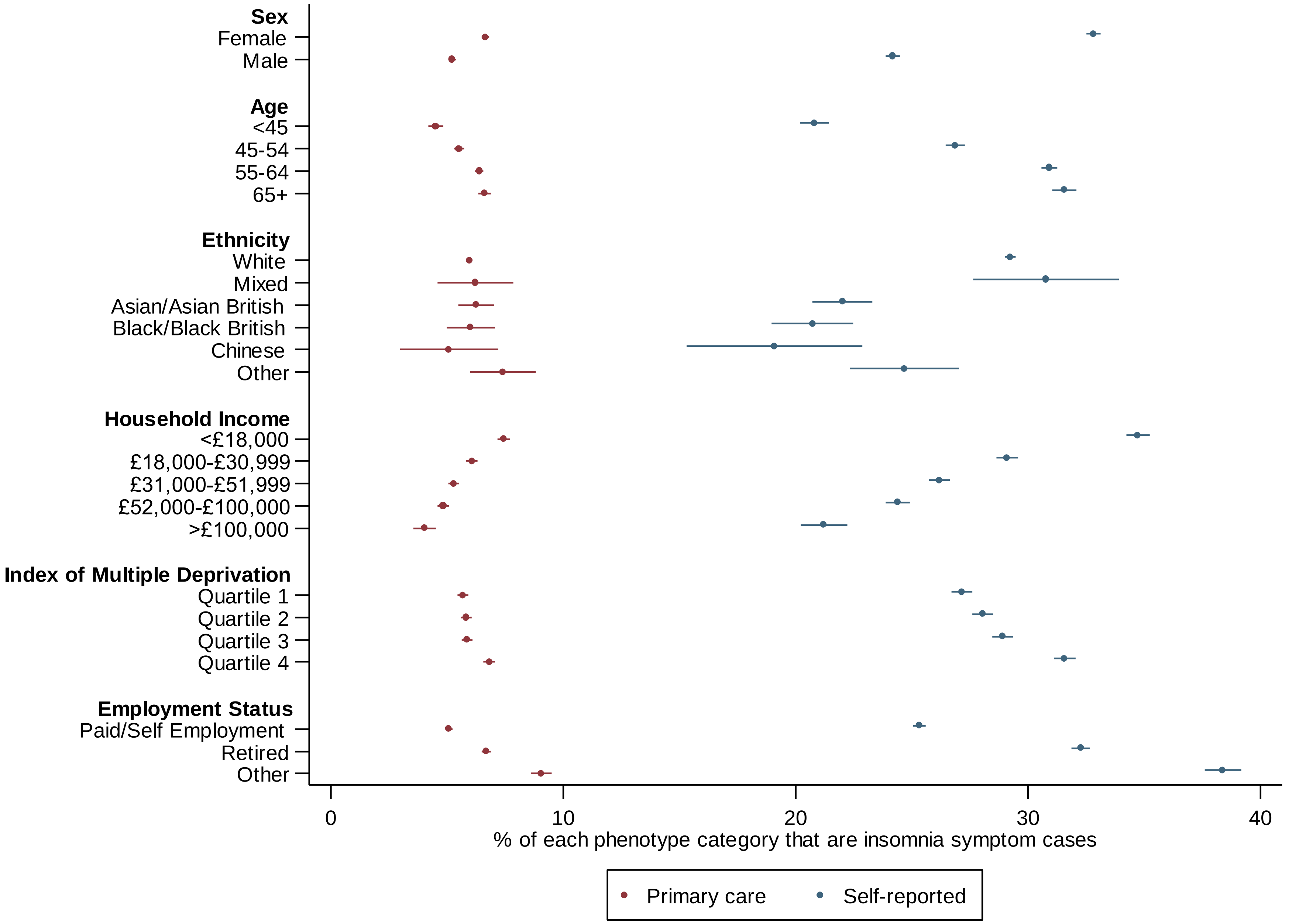

FIGURE S2 Coefficient plot of primary care and self-reported insomnia symptom cases stratified by qualifications, household size, living with spouse/partner, population density, sleep duration, chronotype and snoring

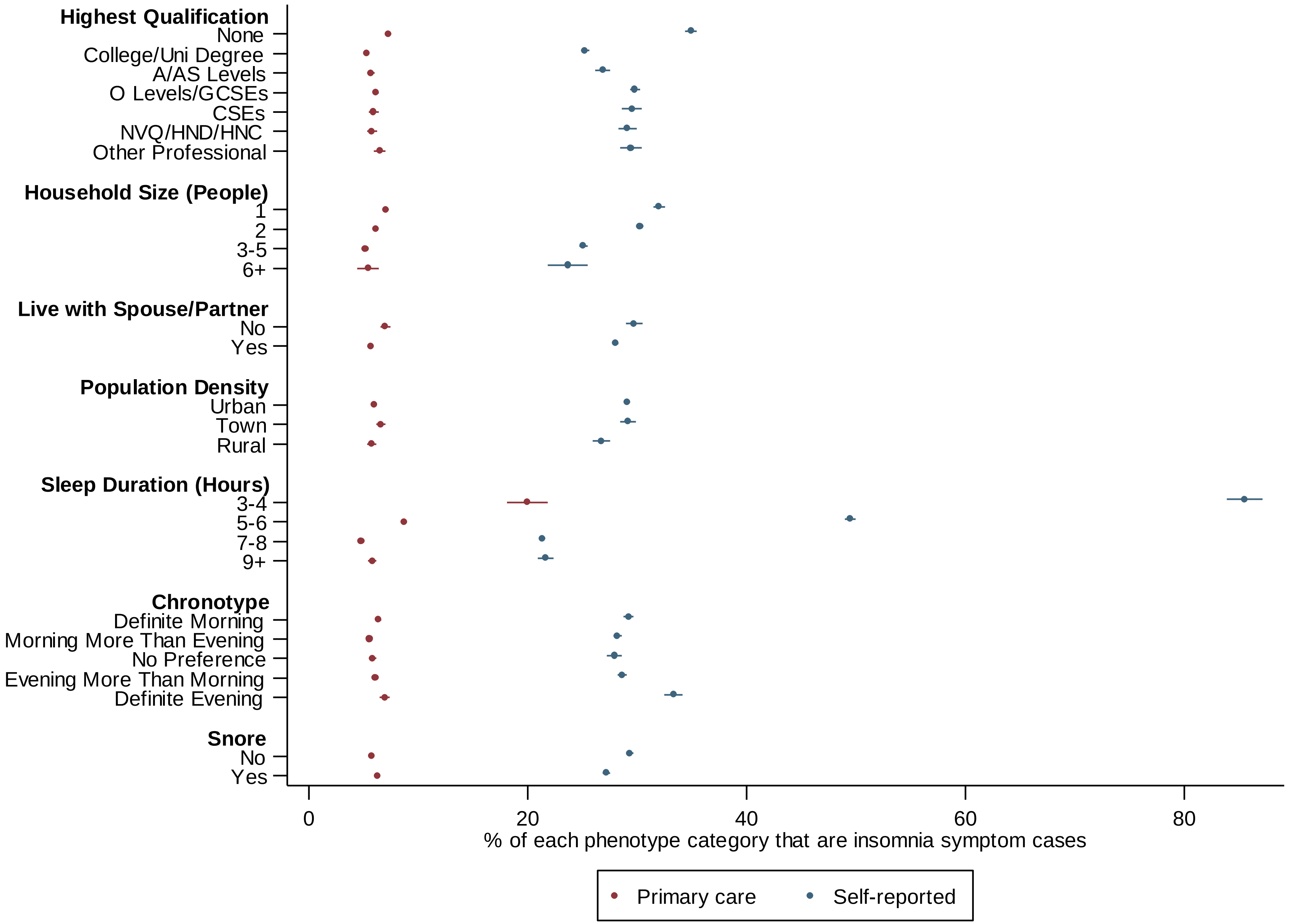

FIGURE S3 Coefficient plot of primary care and self-reported insomnia symptom cases stratified by daytime dozing, daytime napping, ease of getting up in the morning, working night shifts, MET mins/week, and coffee and tea intake.

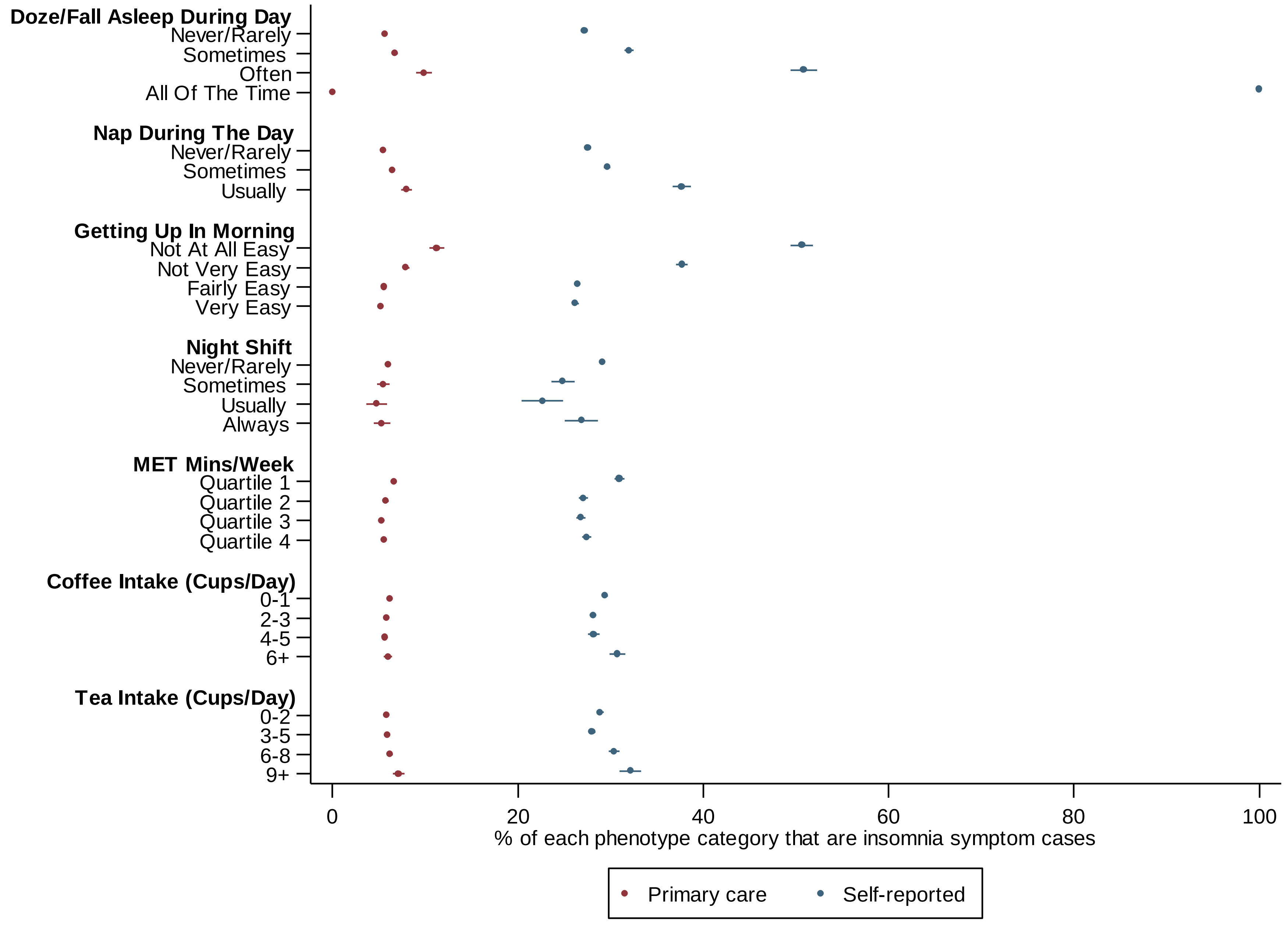

FIGURE S4 Coefficient plot of primary care and self-reported insomnia symptom cases stratified by BMI, risk taking, smoking status, alcohol intake, menopause, depression, worrier and overall health rating

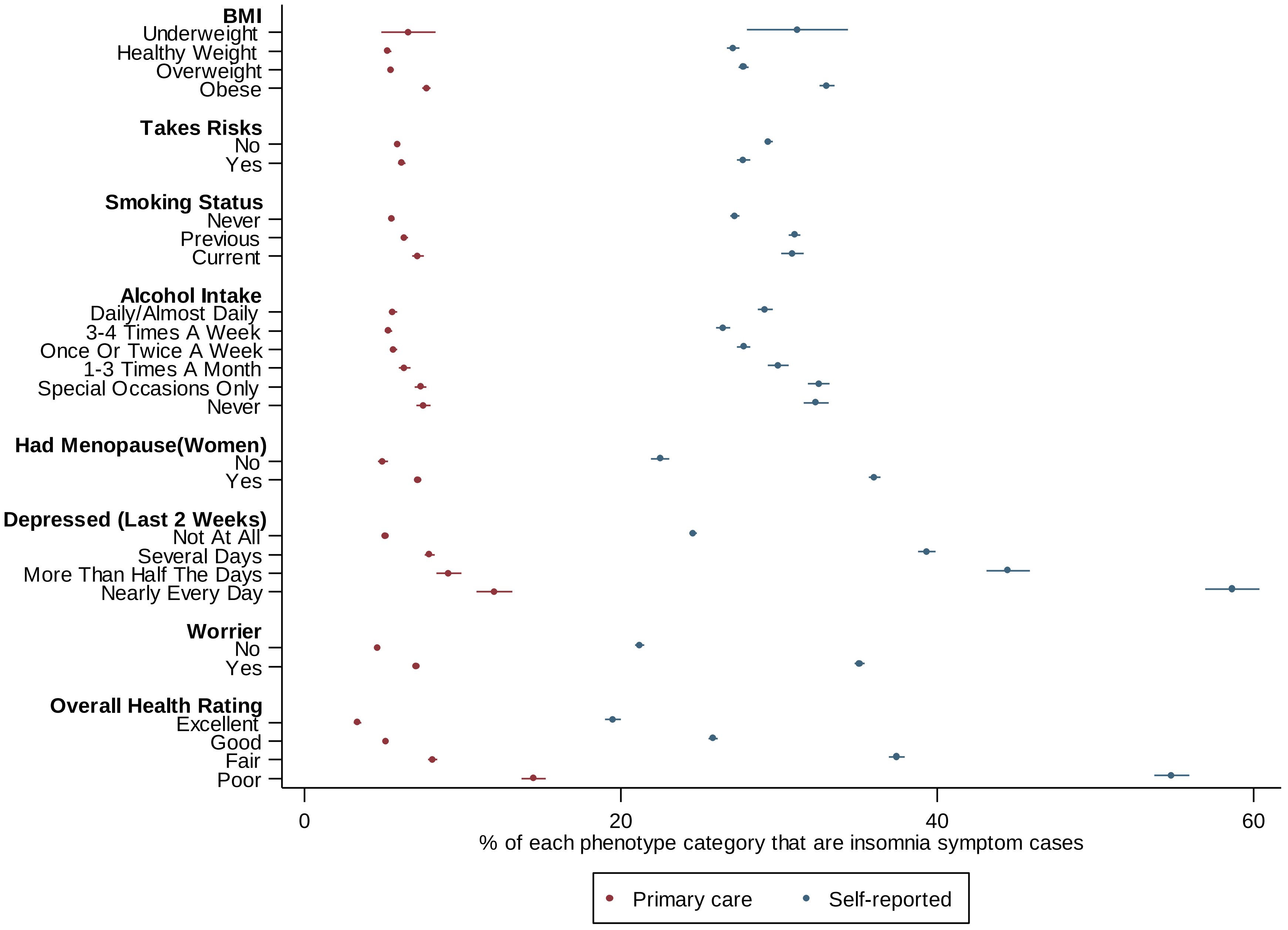

Supplement: online supplemental file 6 [file bmjopen-2023-080479supp006.pdf]
