# Supplementary material for: Insomnia symptom prevalence in England: a comparison of cross-sectional self-reported data and primary care records in the UK Biobank
Source: BMJ Open. 2024 May 7;14(5):e080479. doi: 10.1136/bmjopen-2023-080479 (PMC11086527; doi:10.1136/bmjopen-2023-080479)
Supplement: online supplemental file 7 [file bmjopen-2023-080479supp007.pdf]

Tables S5-S10 Sensitivity Analyses Cross-Tabulations

TABLE S5 Cross-tabulation of Self-Reported Insomnia Symptom Cases and Primary Care Insomnia Symptom Cases (defined as having an insomnia Read code in the 12 months prior to baseline).

**Primary care insomnia symptom case**		**Self-reported insomnia symptom case**		
		No	Yes	Total
No	Frequency	115,570	46,269	161,839
	Row %	71.4	28.6	100.0
	Column %	99.3	97.8	98.8
Yes	Frequency	844	1,065	1,909
	Row %	44.2	55.8	100.0
	Column %	0.7	2.3	1.2
Total	Frequency	116,414	47,334	163,748
	Row %	71.1	28.9	100.0
	Column %	100.0	100.0	100.0

TABLE S6 Cross-tabulation of Self-Reported Insomnia Symptom Cases and Primary Care Insomnia Symptom Cases (defined as having an insomnia Read code in the four weeks prior to baseline).

**Primary care insomnia symptom case**		**Self-reported insomnia symptom case**		
		No	Yes	Total
No	Frequency	116,342	47,208	163,550
	Row %	71.1	28.9	100.0
	Column %	99.9	99.7	99.9
Yes	Frequency	72	126	198
	Row %	36.4	63.6	100.0
	Column %	0.1	0.3	0.1
Total	Frequency	116,414	47,334	163,748
	Row %	71.1	28.9	100.0
	Column %	100.0	100.0	100.0

TABLE S7 Cross-tabulation of Self-Reported Insomnia Symptom Cases and Primary Care Insomnia Symptom Cases (defined as having an insomnia Read code prior to baseline and a prescription for a hypnotic within 90 days of the Read code).

**Primary care insomnia symptom case**		**Self-reported insomnia symptom case**		
		No	Yes	Total
No	Frequency	115,134	45,591	160,725
	Row %	71.6	28.4	100.0
	Column %	98.9	96.3	98.2
Yes	Frequency	1,280	1,743	3,023
	Row %	42.3	57.7	100.0
	Column %	1.1	3.7	1.9
Total	Frequency	116,414	47,334	163,748
	Row %	71.1	28.9	100.0
	Column %	100.0	100.0	100.0

TABLE S8 Cross-tabulation of Self-Reported Insomnia Symptom Cases and Primary Care Insomnia Symptom Cases (defined as having an insomnia Read code in the 12 months prior to baseline and a prescription for a hypnotic within 90 days of the Read code).

**Primary care insomnia symptom case**		**Self-reported insomnia symptom case**		
		No	Yes	Total
No	Frequency	116,224	46,981	163,205
	Row %	71.2	28.8	100.0
	Column %	99.8	99.3	99.7
Yes	Frequency	190	353	543
	Row %	35.0	65.0	100.0
	Column %	0.2	0.8	0.3
Total	Frequency	116,414	47,334	163,748
	Row %	71.1	28.9	100.0
	Column %	100.0	100.0	100.0

TABLE S9 Cross-tabulation of Self-Reported Insomnia Symptom Cases and Primary Care Insomnia Symptom Cases (defined as having an insomnia Read code in the four weeks prior to baseline and a prescription for a hypnotic within 90 days of the Read code).

**Primary care insomnia symptom case**		**Self-reported insomnia symptom case**		
		No	Yes	Total
No	Frequency	116,400	47,297	163,697
	Row %	71.1	28.9	100.0
	Column %	99.99	99.92	99.97
Yes	Frequency	14	37	51
	Row %	27.5	72.6	100.0
	Column %	0.01	0.08	0.03
Total	Frequency	116,414	47,334	163,748
	Row %	71.1	28.9	100.0
	Column %	100.0	100.0	100.0

TABLE S10 Cross-tabulation of Self-Reported Insomnia Symptom Cases and Primary Care Insomnia Symptom Cases (defined as having a hypnotic prescription prior to baseline).

**Primary care insomnia symptom case**		**Self-reported insomnia symptom case**		
		No	Yes	Total
No	Frequency	105,940	39,249	145,189
	Row %	73.0	27.0	100.0
	Column %	91.00	82.9	88.7
Yes	Frequency	10,474	8,085	18,559
	Row %	56.4	43.6	100.0
	Column %	9.00	17.1	11.3
Total	Frequency	116,414	47,334	163,748
	Row %	71.1	28.9	100.0
	Column %	100.0	100.0	100.0
