# Supplementary material for: Insomnia symptom prevalence in England: a comparison of cross-sectional self-reported data and primary care records in the UK Biobank
Source: BMJ Open. 2024 May 7;14(5):e080479. doi: 10.1136/bmjopen-2023-080479 (PMC11086527; doi:10.1136/bmjopen-2023-080479)
Supplement: Supplementary file 8 [file bmjopen-2023-080479.reviewer_comments.pdf]

PEER REVIEW HISTORY

BMJ Open publishes all reviews undertaken for accepted manuscripts. Reviewers are asked to complete a checklist review form (**http://bmjopen.bmj.com/site/about/resources/checklist.pdf**) and are provided with free text boxes to elaborate on their assessment. These free text comments are reproduced below.

ARTICLE DETAILS

**TITLE (PROVISIONAL)**	Insomnia symptom prevalence in England: A comparison of cross-sectional self-reported data and primary care records in the UK Biobank
**AUTHORS**	de Lange, Melanie; Richmond, Rebecca; Eastwood, Sophie V.; Davies, Neil

VERSION 1 – REVIEW

**REVIEWER**	Appleton, Sarah<br>Flinders University
**REVIEW RETURNED**	30-Nov-2023

**GENERAL COMMENTS**	This is a well written manuscript in a very large cohort study of largely white adults that confirms other studies showing that administrative databases and EHR are relatively poor for identifying sleep disorders that go largely underdiagnosed. In relation to insomnia symptoms, self-report of symptoms and questionnaires are more valid ways to identify caseness, including current definitions of chronic insomnia according to the DSM-5 and ICSD-3. The primary care setting is not often amenable to addressing sleep health sue to time constraints and there is evidence that primary care providers do not ask or engage in discussions about sleep very often. In regard to this, the conclusion of the abstract that “only a small proportion of individuals experiencing insomnia symptoms present to primary care” is not necessarily accurate. People affected do present but are simply not asked about their sleep health.<br>I have only one main concern about this MS which is the extensive list of read codes used, many of which are not relevant to insomnia and should be excluded. Classification using appropriate codes is likely to generate a smaller sample and identify a greater mis-match between self-reported symptoms and primary care records. Given the rarity of some of those 181 diagnoses/codes it is unclear what the influence of some of these are. OSA which is very common but frequently undiagnosed is comorbid with insomnia and affects 3-5% of those in epidemiological studies and this may partly explain the observed association with snoring with primary care insomnia symptoms.<br>The low representation of CALD participants in the sample should be highlighted given sleep issues are common in these groups who may experience socioeconomic disadvantage know to be a strong correlate of insomnia symptoms. The findings are likely to underestimate insomnia symptom prevalence and the degree of mis-match.

VERSION 1 – AUTHOR RESPONSE

Reviewer: 1
Dr. Sarah Appleton, Flinders University
Comments to the Author:
This is a well written manuscript in a very large cohort study of largely white adults that confirms other studies showing that administrative databases and EHR are relatively poor for identifying sleep disorders that go largely underdiagnosed. In relation to insomnia symptoms, self-report of symptoms and questionnaires are more valid ways to identify caseness, including current definitions of chronic insomnia according to the DSM-5 and ICSD-3. The primary care setting is not often amenable to addressing sleep health sue to time constraints and there is evidence that primary care providers do not ask or engage in discussions about sleep very often. In regard to this, the conclusion of the abstract that "only a small proportion of individuals experiencing insomnia symptoms present to primary care" is not necessarily accurate. People affected do present but are simply not asked about their sleep health.

The abstract conclusion has been updated to:
"Only a small proportion of individuals experiencing insomnia symptoms have an insomnia symptom Read code in their primary care record…. Further studies should replicate our findings in other populations and examine ways to increase discussions about sleep health in primary care."

The final sentence of our discussion has been updated to read:
"Further studies should replicate our findings in other populations and examine the best ways to increase discussions about sleep health in primary care."

I have only one main concern about this MS which is the extensive list of read codes used, many of which are not relevant to insomnia and should be excluded. Classification using appropriate codes is likely to generate a smaller sample and identify a greater mis-match between self-reported symptoms and primary care records. Given the rarity of some of those 181 diagnoses/codes it is unclear what the influence of some of these are.

We deliberately used a very broad list of Read codes to define insomnia symptoms in the primary care data. This was so that the list corresponded as closely as possible to the very broad UKB self-report question which doesn't ask about the cause of the insomnia: "Do you have trouble falling asleep at night or do you wake up in the middle of the night?".

Because we were trying to compare the self-report data to the same person's primary care data we wanted our Read code list to capture anyone who would have said yes to the self-report question so that we were comparing like-for-like. We have made this more explicit in the method and highlighted in the discussion that if a narrower code list had been used the prevalence in primary care (& subsequently the overlap between self-report and primary care) would have been even lower:

Method:
"Code list creation
To create our list of Read codes for insomnia symptoms we searched the UK Biobank Read CTV3 code lookup table for codes containing the following strings: *insomn* *sleep* or *wak*. This gave us an initial list of 385 insomnia Read codes. This list was then refined by primary care clinician review. We kept our list of insomnia symptom Read codes broad so that we would capture anyone who answered "Usually" to the non-specific UK Biobank self-report question (which did not ask about the cause of the insomnia symptoms). We therefore included codes involving physical (organic) causes of sleep problems (e.g. asthma, chronic obstructive pulmonary disease, sleep apnea), parasomnias (e.g. sleepwalking) and sleep pattern disturbances. Duplicate codes were removed. This resulted in a final list of 181 Read codes (Table S1). Many Read terms for insomnia do not differentiate between diagnoses and symptoms, therefore we did not examine the prevalence of diagnoses alone[29]."

Discussion:
"We used a very broad list of Read codes to define insomnia symptoms in primary care. A narrower Read code list would produce a lower prevalence of primary care insomnia symptoms, as well as a smaller overlap between primary care and self-report insomnia symptom cases."

Our code list was also broad so that our findings could be compared to the statistic that a third of the population suffer from insomnia symptoms ,which comes from studies where the underlying cause of the insomnia is not considered.

The introduction of the paper has been updated to read:
"Estimates of insomnia prevalence differ depending on the definition used. A review of 50 studies from different countries found that around a third of the general population have insomnia symptoms **(defined as difficulty initiating/maintaining sleep or non-restorative sleep, regardless of the underlying cause). In addition,** 9-15% suffer from daytime consequences of insomnia, 8–18% are dissatisfied with their sleep and 6% meet the criteria for an insomnia diagnosis[15].

The start of the discussion has been updated to read:

"We used data from the UK Biobank to compare the prevalence of self-reported insomnia symptoms to the prevalence indicated by linked primary care records. **We included all insomnia symptoms, regardless of the underlying cause."**

OSA which is very common but frequently undiagnosed is comorbid with insomnia and affects 3-5% of those in epidemiological studies and this may partly explain the observed association with snoring with primary care insomnia symptoms.

Thank you for this very good point. We have updated the text of our discussion to include:

"We also found that snoring predicted being a primary care insomnia symptoms case. However, the opposite was true of self-reported cases (i.e. self-reported insomniacs were less likely to report snoring). This suggests that snoring may be a key risk factor for prompting those with insomnia to visit their primary care doctor to discuss their sleep. The discrepancy in snoring between self-report and primary care-identified insomnia cases may be due to the fact that the UK Biobank question about snoring actually relates to participants' partners' report of their snoring ("Does your partner or a close relative or friend complain about your snoring?"). Because snoring can affect the partner's sleep[35] and mental health[36], their partner may encourage them to seek medical help, even if they themselves do not recognise that they snore. However, this was not supported by our analysis which suggests that primary care insomnia cases are more likely to be living alone. An alternative explanation is the fact snoring can be a sign of obstructive sleep apnoea, and that insomnia and obstructive sleep apnoea often occur together. For example, 40-60% of those with sleep apnoea also have insomnia symptoms. This has led to the identification of a new disorder: "comorbid insomnia and obstructive sleep apnoea (COMISA). However, more research is needed to fully understand this disorder[37]. It may be that those with insomnia are more likely to be diagnosed with this by a GP if they also have sleep apnoea because sleep apnoea itself is more likely to impair daytime functioning, such as presenting with daytime drowsiness and difficulty driving, so GPs are more likely to diagnose and treat it."

The low representation of CALD participants in the sample should be highlighted given sleep issues are common in these groups who may experience socioeconomic disadvantage know to be a strong correlate of insomnia symptoms. The findings are likely to underestimate insomnia symptom prevalence and the degree of mis-match.

We have updated the text of discussion to the following:
"Our study also has several limitations. Firstly, the UK Biobank is not representative of the UK population, with participants more likely to be female, healthier, older and live in less socioeconomically deprived areas than non-participants[27]. If having insomnia also affects participation in the UK Biobank, then this may have caused selection bias in our estimates of insomnia prevalence. This is possible, as our study only included a small proportion of ethnic minority participants, and ethnic minorities are more likely to experience sleep problems and may experience socioeconomic disadvantage, itself a correlate of insomnia symptoms[38]."

Reviewer: 1

Competing interests of Reviewer: none

VERSION 2 – REVIEW

**REVIEWER**	Appleton, Sarah<br>Flinders University
**REVIEW RETURNED**	22-Mar-2024

**GENERAL COMMENTS**	I have two additional comments regarding the revision.<br>Firstly given the number of read codes that do not directly relate to insomnia as it is understood as a clinical sleep disorder, I strongly suggest that the following justification (second sentence of the Discussion) be worded from "We included all insomnia symptoms, regardless of the underlying cause". to read "We included all insomnia symptoms and sleep disturbance , regardless of the underlying cause.<br>Secondly, please use patient centered language and people with insomnia in preference to the term 'insomniacs'.

VERSION 2 – AUTHOR RESPONSE

I have two additional comments regarding the revision.
Firstly given the number of read codes that do not directly relate to insomnia as it is understood as a clinical sleep disorder, I strongly suggest that the following justification (second sentence of the Discussion) be worded from "We included all insomnia symptoms, regardless of the underlying cause". to read "We included all insomnia symptoms and sleep disturbance, regardless of the underlying cause.

The text of this sentence in the discussion now reads:

“We included all insomnia symptoms **and sleep disturbance**, regardless of the underlying cause.”

Secondly, please use patient-centered language and 'people with insomnia' in preference to the term 'insomniacs'.

The sentence this refers to has been changed from:

“However, the opposite was true of self-reported cases (i.e. self-reported insomniacs were less likely to report snoring).”

To:

However, the opposite was true of self-reported cases (i.e. **people who self-reported having insomnia** were less likely to report snoring).
